# Disease-Associated Mutation A554V Disrupts Normal Autoinhibition of DNMT1

**DOI:** 10.3390/dna3030010

**Published:** 2023-07-13

**Authors:** Rebecca L. Switzer, Zach J. Hartman, Geoffrey R. Hewett, Clara F. Carroll

**Affiliations:** 1 Department of Chemistry, Bucknell University, Lewisburg, PA 17837, USA; 2 Department of Biology, Bucknell University, Lewisburg, PA 17837, USA; 3 Program in Cell Biology/Biochemistry, Bucknell University, Lewisburg, PA 17837, USA

**Keywords:** DNA methylation, epigenetics, DNA methyltransferase, neurodegenerative diseases

## Abstract

DNA methyltransferase 1 (DNMT1) is the enzyme primarily responsible for propagation of the methylation pattern in cells. Mutations in DNMT1 have been linked to the development of adult-onset neurodegenerative disorders; these disease-associated mutations occur in the regulatory replication foci-targeting sequence (RFTS) domain of the protein. The RFTS domain is an endogenous inhibitor of DNMT1 activity that binds to the active site and prevents DNA binding. Here, we examine the impact of the disease-associated mutation A554V on normal RFTS-mediated inhibition of DNMT1. Wild-type and mutant proteins were expressed and purified to homogeneity for biochemical characterization. The mutation increased DNA binding affinity ~8-fold. In addition, the mutant enzyme exhibited increased DNA methylation activity. Circular dichroism (CD) spectroscopy revealed that the mutation does not significantly impact the secondary structure or relative thermal stability of the isolated RFTS domain. However, the mutation resulted in changes in the CD spectrum in the context of the larger protein; a decrease in relative thermal stability was also observed. Collectively, this evidence suggests that A554V disrupts normal RFTS-mediated autoinhibition of DNMT1, resulting in a hyperactive mutant enzyme. While the disease-associated mutation does not significantly impact the isolated RFTS domain, the mutation results in a weakening of the interdomain stabilizing interactions generating a more open, active conformation of DNMT1. Hyperactive mutant DNMT1 could be responsible for the increased DNA methylation observed in affected individuals.

## 1. Introduction

DNA methylation is a critical epigenetic modification that regulates gene expression. In mammals, DNA methylation occurs predominately on cytosine bases within CpG dinucleotides. Proper DNA methylation patterns are important for normal cellular function. Aberrant methylation patterns have been observed in several diseases including cancer and neurodegenerative disorders [1-3].

DNA methylation patterns are established and primarily maintained by a family of proteins known as DNA methyltransferases (DNMTs). Humans have three active enzymes that methylate DNA: DNMT1, DNMT3a, and DNMT3b. These enzymes all catalyze the transfer of a methyl group from the methyl-donating co-factor *S*-adenosylmethionine to the 5-carbon of cytosine. DNA methylation patterns are established during germ cell development by DNMT3a and DNMT3b. For this reason, these isozymes are often referred to as de novo methyltransferases. They are aided by DNMT3L, a catalytically inactive regulatory factor that enhances their activity. Methylation patterns are maintained through multiple rounds of cell division by the maintenance methyltransferase DNMT1. This isozyme exhibits specificity for methylation of hemimethylated DNA, DNA in which only one strand of the duplex contains methylcytosine [1,3]. Increasing evidence suggests that the classical view of de novo and maintenance methyltransferases having separate and distinct roles might be an oversimplification [4]. For example, studies have pointed to the importance of DNMT1 in de novo methylation [5] and to the surprising presence of this isozyme in postmitotic neurons [6].

**Figure 1.**
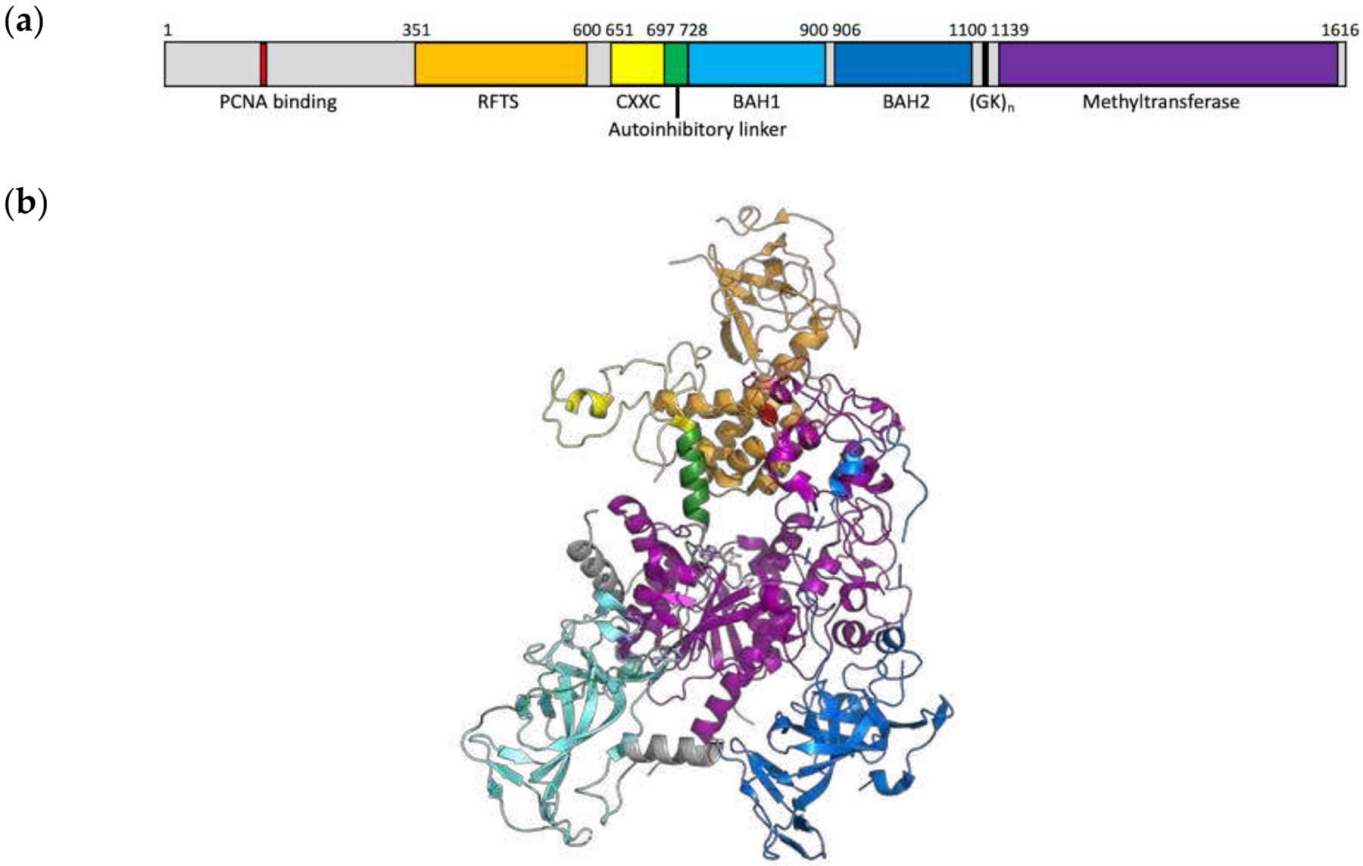
Structure of human DNMT1. (**a**) Domain map of human DNMT1. The protein consists of a C-terminal catalytic domain and an N-terminal regulatory region. The RFTS domain is located in this regulatory region. (**b**) Crystal structure of human DNMT1, amino acids 351-1602 (PDB 4WXX). Domains are colored according to panel (**a**). The inhibitory RFTS domain is bound to the methyltransferase domain. A554 (red spheres) is found in the C lobe of the RFTS domain, with its side chain pointed into the hydrophobic core.

DNMT1 is a large multidomain protein consisting of a C-terminal methyltransferase domain and an N-terminal regulatory region connected by glycine-lysine repeats (Figure 1a). The N-terminal region of the protein is critical for proper targeting and regulation of DNMT1. The regulatory region consists of several domains including the replication foci targeting sequence (RFTS) domain, the CXXC domain, and two bromo-adjacent homology (BAH) domains [3]. Understanding how these regulatory regions work in concert to control DNMT1 activity is a long-standing topic of interest in the field. This work is focused on the role of the RFTS domain. Initially, this domain was reported to target DNMT1 to the replication fork [7]. More recently, biochemical studies revealed that the RFTS domain is an endogenous inhibitory domain, competing with DNA for binding to the active site of DNMT1 [8]. Subsequently, crystal structures of RFTS-containing DNMT1 have shown the RFTS domain bound to the methyltransferase domain (Figure 1b). The RFTS domain consists of two lobes, with the N lobe primarily consisting of beta sheets and the C lobe consisting of a helical bundle. The interdomain interaction is stabilized by hydrogen bonds between residues in the C lobe of the RFTS domain and residues in the methyltransferase domain [9,10]. Targeted mutagenesis of residues involved in these stabilizing hydrogen bonds was shown to increase DNA methylation activity in vitro and in cell culture [11]. Thus, the RFTS domain must be removed from the active site in order for the enzyme to bind and methylate DNA efficiently. Proteins that directly interact with the RFTS domain have been identified, such as ubiquitin-like, containing PHD and RING finger domains 1 (UHRF1) [12,13], and modified histone H3 tails [14-16]. These regulatory protein–protein interactions are proposed to localize DNMT1 to specific sites in the genome and activate the enzyme for catalysis by relieving RFTS-mediated autoinhibition.

Over the past decade, several mutations in *DNMT1* have been reported that result in adult-onset neurodegenerative disorders in humans [17-22]; all of these mutations are germline dominant and occur in the regulatory RFTS domain. The mutations are linked to the development of hereditary sensory and autonomic neuropathy type 1E (HSAN1E) and autosomal dominant cerebellar ataxia, deafness, and narcolepsy (ADCA-DN). These two disorders have many overlapping clinical features including hearing loss and mild to severe neuropathy [22]. The mutated residues associated with HSAN1E development are found throughout the RFTS domain, while the mutated residues associated with ADCA-DN development are found clustered in the C lobe of the RFTS domain [1]. With important intra- and intermolecular regulatory interactions facilitated by the RFTS domain, these disease-associated mutations could potentially impact localization, activation, and/or autoinhibition of DNMT1.

Methylation profiling has shown drastic changes to the normal DNA methylation pattern in affected individuals [23,24]. However, due to a lack of biochemical information, the exact role(s) these mutations play in disease development remains largely unclear. We recently showed that ADCA-DN-associated mutations G589A and V590F relieve normal RFTS-mediated inhibition of DNMT1, leading to hyperactive enzymes [25]. Here, we report the impact of A554V, another mutation associated with ADCA-DN formation, on the autoinhibition of DNMT1. We have recombinantly expressed and purified A554V DNMT1 and RFTS domain to investigate the impact of this mutation on autoinhibition. The mutant protein exhibits increased DNA binding affinity and increased methylation activity. In addition, changes in the circular dichroism (CD) spectrum and relative thermal stability of the mutant protein support the finding that this mutation also weakens normal RFTS-mediated inhibition of DNMT1, generating a hyperactive mutant enzyme.

## 2. Materials and Methods

### 2.1. Expression Constructs and Site-Directed Mutagenesis

Expression constructs for truncated forms of human DNMT1 were previously created [8]. Wild-type expression constructs and primers containing the desired mutation (5′-CCCTCCTGCGACACGTGCAGTTTGTGGTGGAG-3′ and 5′-CTCCACCACAAACTGCA-CGTGTCGCAGGAGGG-3′) were used to generate mutant plasmids using the Agilent QuikChange XL site-directed mutagenesis kit according to the manufacturer’s instructions. A554V was introduced into both RFTS-containing DNMT1 (amino acids 351–1616) and the RFTS domain (amino acids 351–600). Mutations were confirmed by DNA sequencing.

### 2.2. Protein Expression and Purification

Following previously published procedures, wild-type and mutant proteins and domains were expressed in Rosetta 2(DE3) pLysS competent cells [26]. All proteins and domains contain an N-terminal histidine tag. As such, proteins were initially purified from soluble cell lysate by metal affinity chromatography. Wild-type and A554V RFTS-containing DNMT1 were further purified using a 5 mL HiTrap Heparin HP column (Cytiva Life Sciences, Marlborough, MA, USA) as previously described [26]. To obtain highly pure protein for CD analysis, the RFTS-containing DNMT1 proteins were then further purified by size-exclusion chromatography. Fractions from the Heparin purification that contained RFTS-containing DNMT1 were concentrated to 5 mL and loaded onto a HiLoad 16/600 Superdex 200 column (Cytiva Life Sciences) equilibrated in 20 mM HEPES (pH 8), 500 mM NaCl, and 5% glycerol. Protein was eluted with 1.5 column volumes (CVs) of the same buffer. Fractions containing RFTS-containing DNMT1 were pooled, concentrated, and stored in 40% (v/v) glycerol at −80 °C. Following metal-affinity purification, wild-type and A554V RFTS domains were further purified by size-exclusion chromatography using a HiLoad Superdex 75 column (Cytiva Life Sciences) as previously described [25]. Fractions containing RFTS domain were pooled, concentrated, and stored in 40% (*v*/*v*) glycerol at −80 °C. Proteins were quantified using A_280_ and calculated extinction coefficients. Size-exclusion chromatograms and sodium dodecyl sulfate (SDS)-polyacrylamide gel electrophoresis (PAGE) of pure proteins and domains are shown in Figures S1 and S2.

### 2.3. Fluorescence Polarization

DNA binding affinity was measured by fluorescence polarization using a fluorescently tagged 18 base pair hemimethylated duplex DNA as previously described [25]. In short, the concentration of RFTS-containing DNMT1 was varied as DNA concentration was held constant at 100 nM. Triplicate data were collected at 28 °C in a BioTek Synergy plate reader equipped with a fluorescence polarization cube. Data were fit to the hyperbolic binding equation (Equation (1)) in Kaleidagraph (Synergy Software, version 5.01), where P is polarization, $P_{f}$ is the polarization of free DNA, and $P_{b}$ is the polarization of bound DNA, to determine the $K_{d}$.


(1) $$P=P_{f}+\left[\left(P_{b}-P_{f}\right)\frac{\left[DNMT1\right]}{K_{d}+[DNMT1]}\right]$$

Polarization values were normalized and averaged for visualization.

### 2.4. DNA Methylation Assay

Activity of RFTS-containing DNMT1 was assessed using a previously described endonuclease-coupled DNA methylation assay [25]. Assays contained 1 μM 32 base pair hemimethylated duplex DNA, 100 μM *S*-adenosylmethionine, and 0.5 μM RFTS-containing DNMT1. Samples were removed and flash frozen 2, 8, and 20 min after enzyme addition, subsequently digested with Sau3A1 (New England BioLabs, Inc., Ipswich, MA, USA) and separated on 18% PAGE. The gel was stained using ethidium bromide and imaged with a Bio Rad Gel Doc EZ system.

### 2.5. Differential Scanning Fluorimetry (DSF)

DSF was used to examine the thermal stability of RFTS-containing DNMT1 proteins as previously described [25]. Assays contained 0.5 μM RFTS-containing DNMT1 and 5X SYPRO Orange (Invitrogen, Thermo Fisher Scientific, Waltham, MA, USA). Data were collected in a Roche LightCycler 96 real-time PCR instrument using the VIC channel. Fluorescence data were fitted to the Boltzmann equation to determine the observed melting temperature (T_m_). Fluorescence data were normalized for visualization.

### 2.6. Circular Dichroism (CD) Spectroscopy

CD data were recorded on a Jasco J-1500 spectrometer using a 0.1 cm path length cuvette. Spectral accumulation parameters included a scanning rate of 50 nm/min with a 1.0 nm bandwidth over a wavelength range of 190–260 nm; spectra were initially collected at 25 °C. Each spectrum at 25 °C was obtained from an average of 3 scans, corrected for buffer contributions, and smoothed using the Jasco Spectra Manager software, version 2. Secondary structure analysis of the resulting CD spectra was performed using BeStSel (http://bestsel.elte.hu, accessed on 4 April 2023) [27,28]. All data fits resulted in RMSD ≤ 0.043. Spectra were also collected at increasing temperatures using the same parameters. For these spectra, a single scan was collected at 25, 35, 45, 55, 65, and (for RFTS-containing DNMT1 only) 75 °C. Thermal stability of the proteins and domains was investigated by varying the temperature using a ramp rate of 1 °C per minute. Changes in CD signal at 207 nm or 198 nm from 25 to 75 °C were recorded for RFTS-containing DNMT1. Changes in the CD signal at 198 nm or 200 nm from 25 to 65 °C were recorded for RFTS domains. Resultant melting curves were fitted to the Boltzmann equation to determine the observed T_m_. RFTS-containing DNMT1 data were collected at a concentration of 1 μM (0.14 mg/mL), while RFTS domain data were collected at a concentration of 5 μM (0.15 mg/mL) in 20 mM KPi (pH 7.5) buffer.

## 3. Results

Mutations in the RFTS domain of DNMT1 are causal for adult-onset neurodegenerative disorders [17-22]. Here, we investigate the impact of an ADCA-DN-associated mutation—A554V—on RFTS-mediated autoinhibition of DNMT1. We expressed this mutant protein in *Escherichia coli* as an N-terminally truncated form of human DNMT1 that begins with the RFTS domain, termed RFTS-containing DNMT1 (amino acids 351–1616). Previous work has shown that truncating the extreme N-terminus of DNMT1 does not impact the protein’s behavior in in vitro assays [8,29,30]. We also expressed mutant RFTS domain (amino acids 351–600) in *E. coli*. We were able to obtain pure wild-type and mutant truncated proteins and protein domains for biochemical characterization (Figures S1 and S2).

### 3.1. Mutation Increases DNA Binding Affinity

**Figure 2.**
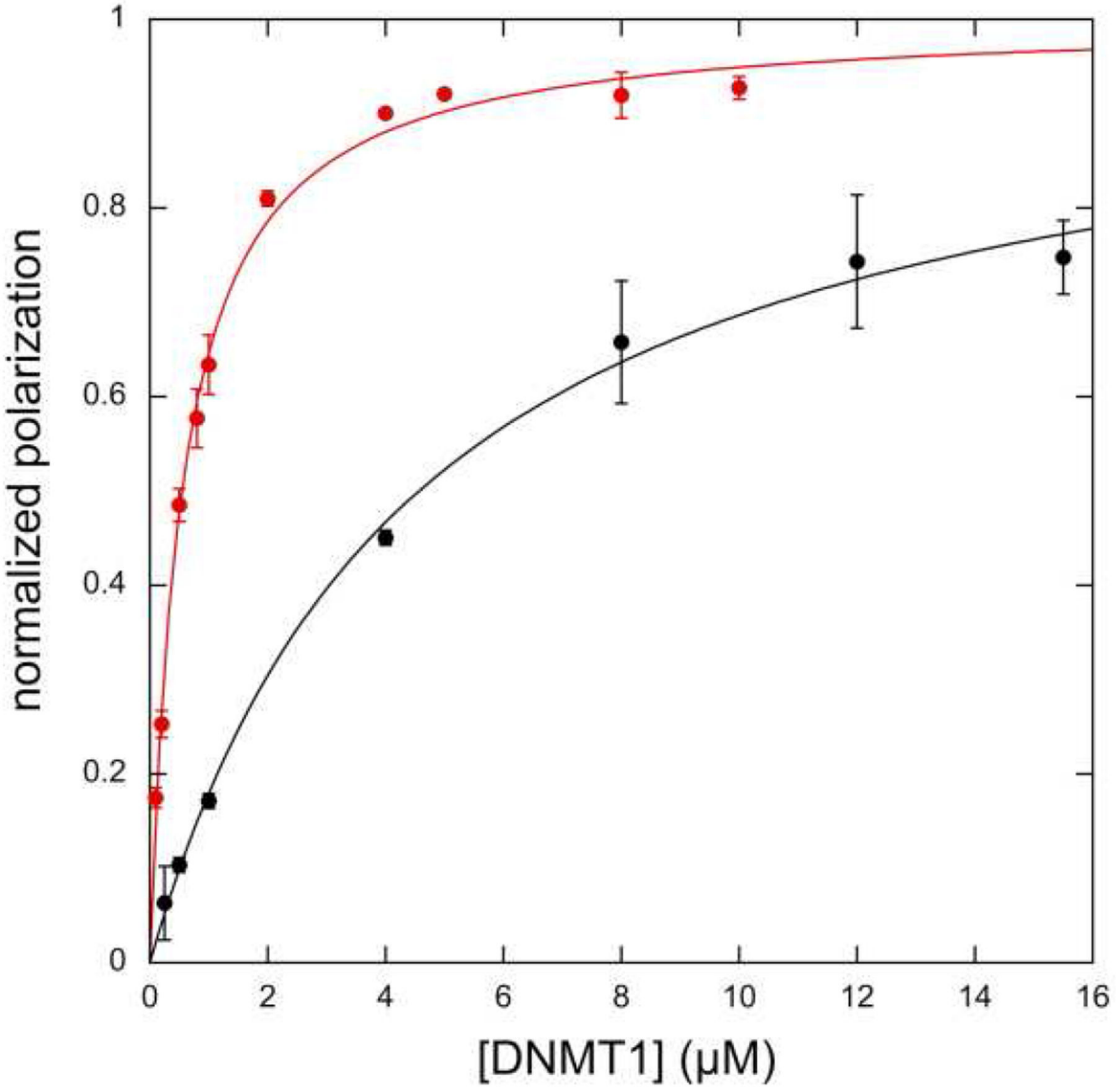
DNA binding of RFTS-containing DNMT1 using fluorescence polarization. Polarization values from triplicate assays were normalized and averaged; error bars represent standard deviation. A554V RFTS-containing DNMT1 (red) binds DNA 8-fold tighter than wild-type RFTS-containing DNMT1 (black).

The RFTS domain of DNMT1 is an endogenous inhibitory domain that competes with DNA for binding to the active site [8]. We were interested in determining if the disease-associated mutation A554V affects the normal function of the RFTS domain. To this end, we determined the DNA binding affinity of wild-type and A554V RFTS-containing DNMT1. Fluorescence polarization of an 18-base-pair hemimethylated duplex DNA labeled with fluoroscein was measured. Binding of this relatively small DNA by the much larger DNMT1 to generate a proteine•DNA complex would be expected to increase the observed polarization value. Titrating a fixed concentration of DNA with increasing concentrations of RFTS-containing DNMT1 does lead to an increase in the observed polarization value (Figure 2). Fitting the data obtained to Equation (1) allows for the binding affinity for DNA to be determined. Wild-type RFTS-containing DNMT1 bound the short duplex DNA with a K_d_ of 4.6 ± 0.5 μM. This value is in good agreement with previously published data showing the DNA binding affinity for wild-type RFTS-containing DNMT1 was 4.4 μM [25]. Introduction of the disease-associated mutation A554V greatly increased the observed DNA binding affinity (Figure 2). The mutant protein bound DNA with a K_d_ of 0.55 ± 0.05 μM, an eightfold increase in affinity. This drastic increase in DNA binding ability suggests that RFTS-mediated inhibition has been significantly weakened in the mutant enzyme.

### 3.2. Mutation Increases DNA Methylation Activity

**Figure 3.**
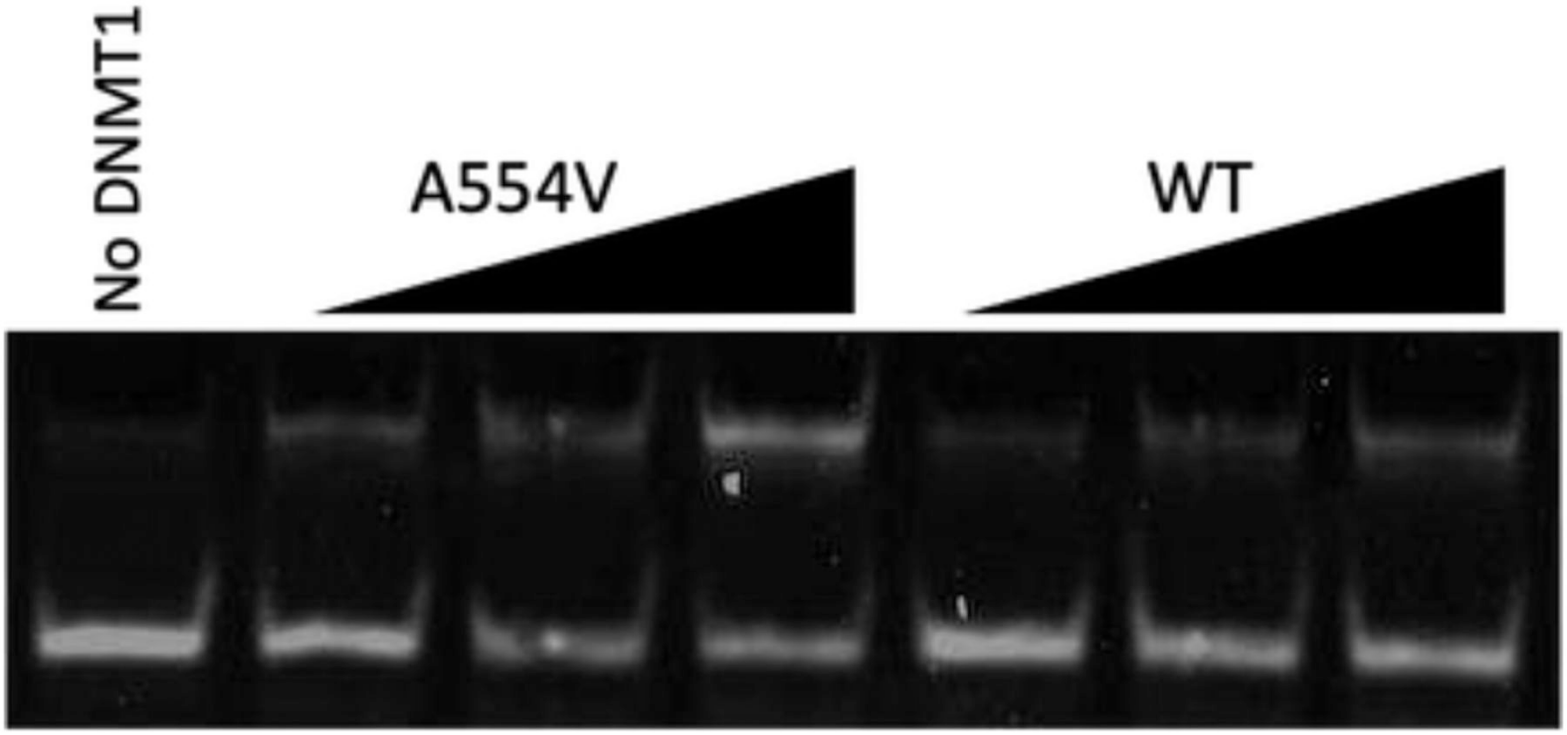
DNA methylation activity of RFTS-containing DNMT1. Wild-type (WT) or A554V RFTS-containing DNMT1 were added to assays containing a hemimethylated DNA substrate and *S*-adenosylmethionine. Methylation of the substrate DNA protects against Sau3A1 cleavage. Samples were taken throughout a 20 min incubation at 37 °C. Digested samples were separated by gel electrophoresis. For each enzyme, 2, 8, and 20 min samples are shown. A control lacking DNMT1 is shown in the first lane.

The RFTS domain is a potent inhibitor of the DNA methylation activity of DNMT1; the presence of the RFTS domain inhibits catalytic efficiency by ~640-fold [8]. With the observed increase in the DNA binding ability of A554V RFTS-containing DNMT1, we next wanted to examine the DNA methylation activity of the mutant protein. To undertake this, we utilized a gel-based endonuclease-coupled DNA methylation assay [25,31]. This assay utilizes the methyl-sensitive restriction endonuclease Sau3A1. Cleavage of the substrate hemimethylated duplex DNA is blocked by full methylation. Thus, DNMT1 activity protects the DNA from cleavage. Separating Sau3A1-digested assay samples by gel electrophoresis allows for the visualization of DNMT1 activity. The addition of wild-type RFTS-containing DNMT1 to assays containing the hemimethylated DNA substrate and *S*-adenosylmethionine results in time-dependent protection from Sau3A1 cleavage, showing the enzyme is active (Figure 3). Under identical assay conditions, the addition of A554V RFTS-containing DNMT1 resulted in more robust protection over time, indicating increased activity in the mutant enzyme. These results suggest that the mutant enzyme is hyperactive and supports the notion that A554V relieves normal RFTS-mediated autoinhibition.

### 3.3. Mutation Impacts Structure and Stability

**Figure 4.**
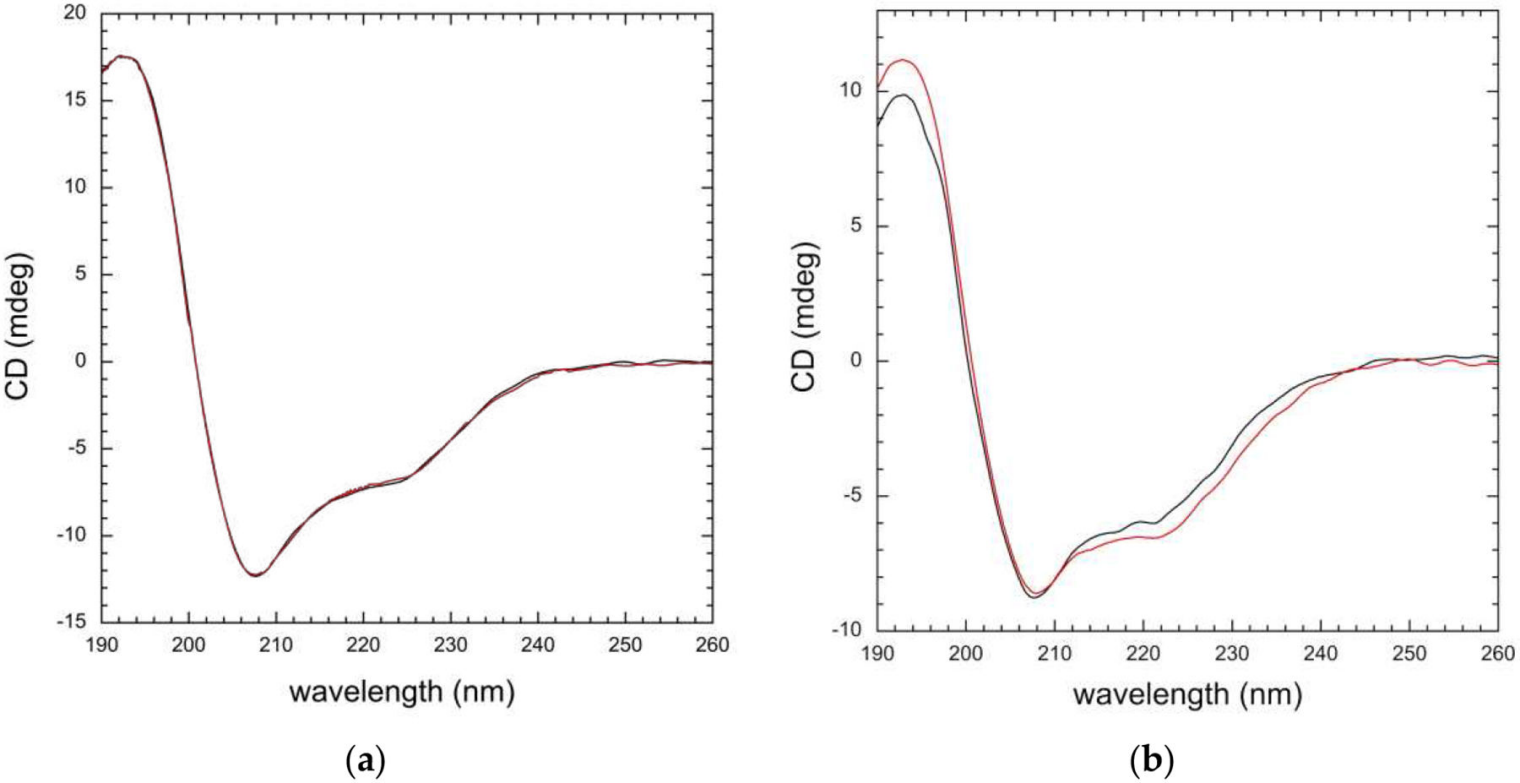
CD analysis of wild-type and A554V proteins at 25 °C. (**a**) Far UV CD spectra of wild-type (black) and A554V (red) RFTS domains (5 μM); (**b**) far UV CD spectra of wild-type (black) and A554V (red) RFTS-containing DNMT1 (1 μM). Spectra shown have been corrected for buffer signal and smoothed.

The biochemical data suggest that A554V disrupts normal RFTS-mediated inhibition of DNMT1 activity. CD spectroscopy was used to examine the impact of the mutation on the structure of DNMT1. The RFTS domain is independently stable and can be expressed in *E. coli* and purified to homogeneity in the absence of the rest of the protein [8]. We first examined wild-type and mutant RFTS domain to determine the impact of the mutation on the structure of the domain itself. Far UV CD spectra of the domains were obtained from 190–260 nm at 25 °C. (Figure 4a). This region mainly reflects electronic transitions of peptide bonds. In the common secondary structural elements found in proteins, the peptide bonds are regularly ordered, leading to characteristic spectral profiles [32]. Thus, CD spectra in this region are largely reporting on the secondary structure of a protein. The RFTS domain is comprised of a zinc-binding motif, a *β*-barrel, and an *α*-helical bundle [8]. The CD spectrum of the RFTS domain exhibits a minimum at 208 nm and a positive peak at 193 nm, characteristics observed in *α*-helical proteins [33]. However, the negative peak typically observed in *α*-helical proteins near 222 nm is severely repressed in the RFTS domain spectrum. Nonetheless, the CD spectra of wild-type and A554V RFTS domains are incredibly similar, suggesting that the structure of the domain was not impacted significantly by the mutation. BeStSel was used to analyze the CD spectra for secondary structure content [27,28]. As expected based on visual inspection of the spectra, wild-type and mutant RFTS domains exhibit similar predicted secondary structure content (Table 1).

We next wanted to examine the impact of the RFTS mutation on the entire protein. To undertake this, we investigated RFTS-containing DNMT1 proteins. DNMT1 is a large multidomain protein known to exist in an inhibited state where the RFTS domain is bound to the methyltransferase domain [1,9,10]. The protein must undergo structural changes that remove the RFTS domain from the active site so that DNA can be methylated. Our biochemical data suggest that A554V weakens the interdomain interaction between the RFTS and methyltransferase domains. We were curious about whether changes in the CD spectrum would be evident in the mutant protein. Interestingly, the far UV CD spectra of wild-type and A554V RFTS-containing DNMT1 exhibit some noticeable differences (Figure 4b). These differences cannot be attributed to concentration differences as the A_280_ of the protein sample in each cuvette was verified to be the same. Moreover, the differences in signal amplitude observed vary across the spectrum. A554V RFTS-containing DNMT1 exhibits a higher signal in the 193 nm peak as compared to the wild-type enzyme, but the minimum at 208 nm is virtually identical between the two proteins. The negative peak near 222 nm is more pronounced in the mutant protein as well. We again used BeStSel to analyze these spectra for secondary structure content [27,28]. The major difference in predicted secondary structure content is in the number of helices (Table 2). A554V RFTS-containing DNMT1 lost ~2% helical content as compared to the wild-type protein. As position 554 is located in the C-terminal *α*-helical bundle of the RFTS domain [9,10], it is possible that mutation of this residue is impacting the structure of these helices. However, similar losses of helical content were not observed in the mutant RFTS domain alone. Instead, it is conceivable that the changes observed in the CD spectrum of the mutant protein are caused by structural changes resulting from loss of the normal interdomain interaction, i.e., loss of autoinhibition is impacting the structure of DNMT1 outside of the RFTS domain itself.

**Figure 5.**
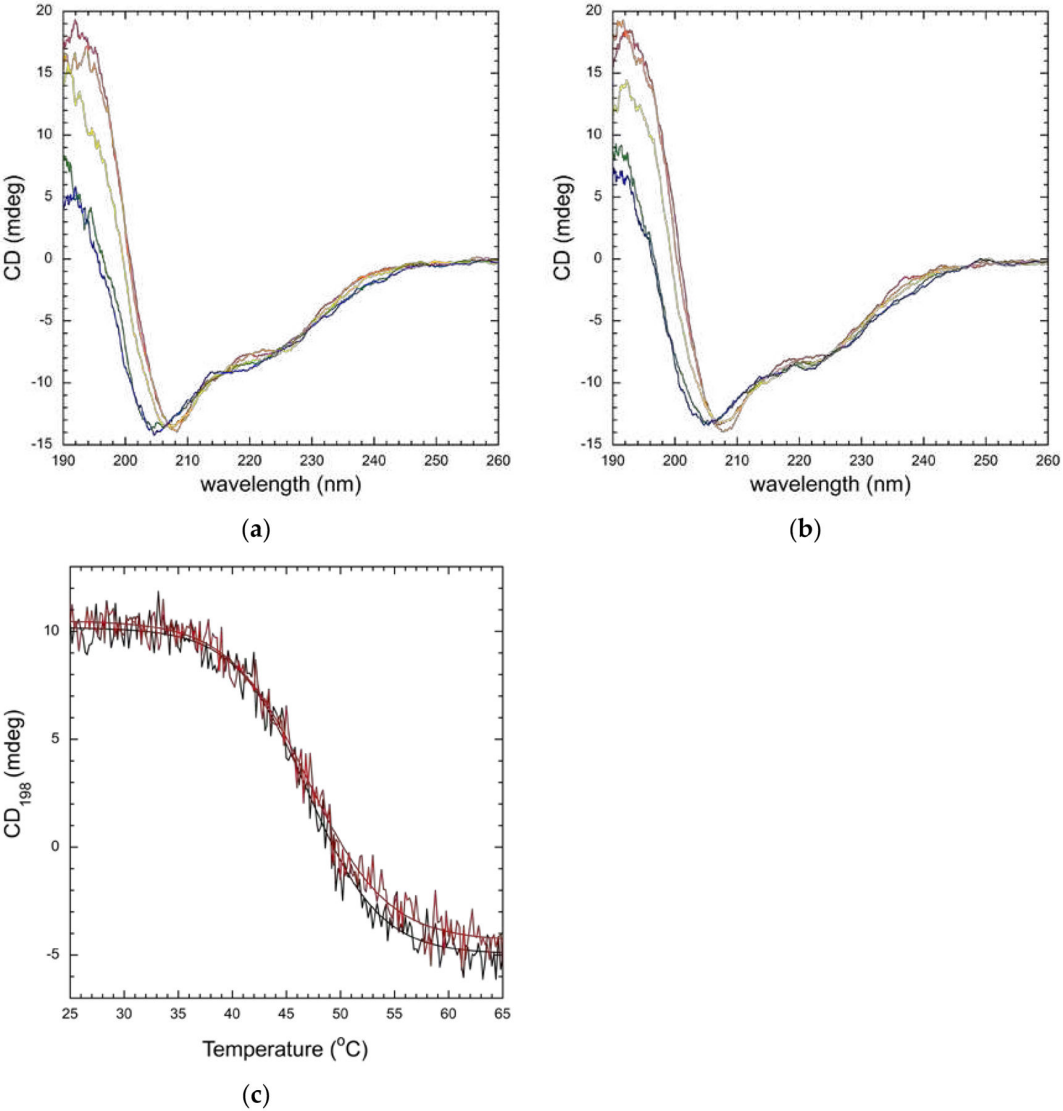
Thermal stability of wild-type and A554V RFTS domain. Temperature-dependent CD spectra of wild-type (**a**) and A554V (**b**) RFTS domain. Spectra from a single scan were collected at 25 (red), 35 (orange), 45 (yellow), 55 (green), and 65 (blue) °C. In both proteins, large changes in CD signal near the 193 nm peak are observed as the protein unfolds; (**c**) thermal stability of wild-type (black) and A554V (red) RFTS domains. CD signal was recorded at 198 nm from 25 to 65 °C. Melting curves were fit to the Boltzmann equation to determine the observed melting temperature (T_m_). Values of 47.0 ± 0.1 and 47.0 ± 0.1 were determined for wild-type and A554V, respectively.

Since the CD signal reports on the structure of a protein, changes in CD spectra are observed as proteins unfold. To examine stability, the proteins were unfolded by increasing the temperature. We first examined changes in the CD spectrum of wild-type and mutant RFTS domain as a function of temperature (Figure 5a,b). As temperature increased, the largest changes in CD signal were observed in the peak at 193 nm. Unfolding of the RFTS domains was not fully reversible as the original CD spectra are not obtained after heating and cooling the proteins. Thermal unfolding of proteins is often irreversible [34], preventing equilibrium thermodynamic analysis. Nonetheless, relative stabilities of wild-type and mutant proteins can be determined when the proteins are examined under the same conditions [35,36]. We decided to monitor RFTS domain unfolding at 198 nm, as this wavelength exhibited better signal-to-noise ratio than 193 nm, and a large change in signal was still observed. As shown in Figure 5c, both wild-type and mutant RFTS domain exhibited very similar monophasic melting. The curves were fit to a sigmoidal equation to determine an observed melting temperature (Tm); comparing the values obtained for wild-type and A554V RFTS domain allows for comparison of thermal stability under these conditions. Fitting the curves, observed T_m_ values of 47.0 ± 0.1 °C were obtained for both wild-type and A554V RFTS domains. Similar observed T_m_ values were obtained in replicate melting experiments and when data were obtained at 200 nm instead of 198 nm (Table S1). Thus, the disease-associated mutation did not impact the thermal stability of the isolated RFTS domain under these conditions.

**Figure 6.**
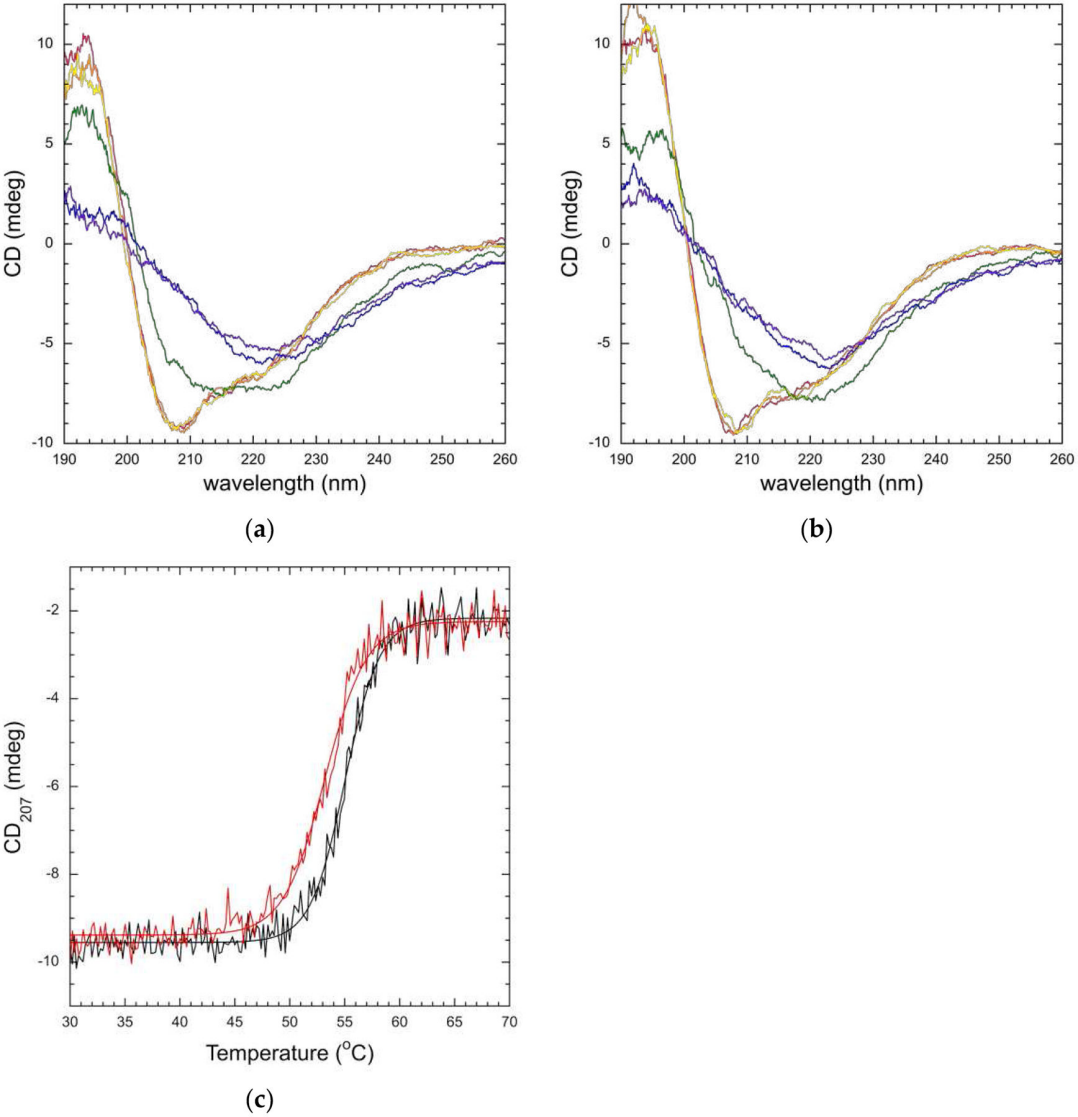
Thermal stability of wild-type and A554V RFTS-containing DNMT1. Temperature-dependent CD spectra of wild-type (**a**) and A554V (**b**) RFTS-containing DNMT1. Spectra from a single scan were collected at 25 (red), 35 (orange), 45 (yellow), 55 (green), 65 (blue), and 75 (purple) °C. In both proteins, large changes in CD signal near the minimum at 208 nm were observed as the protein unfolds; (**c**) thermal stability of wild-type (black) and A554V (red) RFTS-containing DNMT1. CD signal was observed at 207 nm. Melting curves were fit to the Boltzmann equation to determine the observed T_m_. Values of 55.0 ± 0.1 °C and 53.2 ± 0.1 °C were determined for wild-type and A554V, respectively.

We again used changes in CD to track the unfolding of the RFTS-containing DNMT1 proteins. For these proteins, large changes in CD signal were observed at the 193 nm peak and near the minimum at 208 nm as temperature was increased (Figure 6a,b). Similar to the RFTS domains, the unfolding of RFTS-containing DNMT1 was not reversible; the original CD spectrum of the proteins is not obtained after heating and cooling the samples. Thus, we again examined the relative stability of the wild-type and mutant proteins. For RFTS-containing DNMT1, we followed protein denaturation at 207 nm as the signal-to-noise ratio is better in this region of the spectrum, and large changes in signal were observed. Even though DNMT1 is a large multidomain protein, melting curves for wild-type and A554V RFTS-containing DNMT1 were monophasic (Figure 6c). Monophasic melting behavior has been observed previously for large wild-type and mutant DNMT1 constructs [25,37,38]. Fitting the melting curves yielded observed T_m_ values of 55.0 ± 0.1 °C and 53.2 ± 0.1 °C for wild-type and A554V RFTS-containing DNMT1, respectively. Again, the observed T_m_ values were consistent across multiple melting experiments and wavelengths (Table S2). Thus, when using the same experimental parameters (temperature ramp of 1 ^°^C per minute), the RFTS mutation seems to decrease the thermal stability of RFTS-containing DNMT1. Additionally, this loss of stability is also observed in melting curves obtained by differential scanning fluorimetry (DSF) (Figure S3). This data also shows a ~2 °C decrease in the observed T_m_ of the mutant protein. The DSF data were obtained with a slightly faster temperature ramp (2.4 °C per minute), yet the difference in relative stabilities is still observed. Taken together, these data indicate a decrease in relative thermal stability for A554V RFTS-containing DNMT1 even though the mutation did not impact the relative stability of the RFTS domain alone. We suggest this difference is due to a weakening of normal RFTS-mediated inhibition in A554V RFTS-containing DNMT1. Loss of the interdomain stabilizing interactions would make it easier to unfold the mutant protein, even if interactions within the isolated domain were not significantly impacted.

## 4. Discussion

DNMT1 is the enzyme primarily responsible for maintenance of the DNA methylation pattern following cell division. The C-terminal methyltransferase domain is responsible for the catalytic activity of DNMT1; however, the first ~1100 amino acids of the protein are critical for proper regulation, targeting, and activation [3]. DNMT1 is known to play an essential role in replication-linked DNA methylation. Yet, DNMT1 expression has been detected in postmitotic neurons [6,39]. Interestingly, DNA methylation has been shown to be important for adult neurogenesis [40]. Point mutations in DNMT1 have been linked to the development of two adult-onset neurodegenerative disorders: HSAN1E and ADCA-DN [17-22]. These diseases exhibit age-dependent progression and share common clinical manifestations including hearing loss, neuropathy, and cognitive decline [22]. All disease-associated mutations discovered to date map to the RFTS domain, a domain found in the N-terminal regulatory region of DNMT1. The RFTS domain is a critical regulator of DNMT1 activity, both serving as an inhibitory domain that blocks DNA binding and playing important roles in recruitment of DNMT1 to particular sites in the genome and activation of enzyme activity [3].

Here, we report our investigation of the impact of A554V, a mutation associated with development of ADCA-DN, on normal RFTS-mediated autoinhibition of DNMT1. Mutant RFTS-containing DNMT1 and the RFTS domain were able to be expressed as soluble proteins in *E. coli*. The CD spectrum of the A554V RFTS domain was very similar to that of the wild-type protein, indicating that the mutation did not significantly impact the structure of the domain. In addition, A554V RFTS-containing DNMT1 was able to bind and methylate DNA in our studies, indicating that the folding of the larger protein was not severely impacted either. Similarly, previous work has shown that ACDA-DN-associated mutations G589A and V590F, found in the same region of the domain as A554V, did not significantly impact the folding of DNMT1 [25]. This is in stark contrast to studies examining HSAN1E-associated mutations. Expression of full-length DNMT1 containing several different HSAN1E-associated mutations in cell culture resulted in decreases in relative protein levels as compared to wild type. In addition, mutant proteins were found to mislocalize to the cytoplasm and form aggregates, indicating HSAN1E mutations cause decreased stability and the potential misfolding of DNMT1 [17,22]. Furthermore, expression of the RFTS domain containing Y495C or P491Y, mutations associated with HSAN1E formation, generated misfolded, insoluble protein in *E. coli* [17]. Taken together, these data indicate that ADCA-DN-associated mutations result in less severe structural perturbations to the RFTS domain and DNMT1 as a whole.

Normal RFTS-mediated autoinhibition of DNMT1 activity is impacted by the disease-associated mutation. A554V RFTS-containing DNMT1 bound DNA ~8-fold tighter than the wild-type enzyme. This increase in DNA binding affinity is even larger than that reported for the other ADCA-DN-associated mutations. Those mutations resulted in ~2.5–3.5-fold increases in affinity [25], suggesting that A554V is a functionally more severe mutation. As the RFTS domain is a DNA-competitive inhibitor [8], increases in DNA binding affinity are consistent with loss of normal RFTS-mediated inhibition. Previous work has shown that DNMT1 is capable of binding histone H3 tails and that this binding is inhibited by the interdomain interaction between the RFTS domain and the methyltransferase domain [16]. Interestingly, in this study, the ADCA-DN-linked RFTS mutations were shown to increase DNMT1 binding to histone H3 [16], consistent with our finding that these mutations weaken the normal interdomain interaction. Furthermore, A554V RFTS-containing DNMT1 was hyperactive in our endonuclease-coupled DNA methylation assay. Similarly, increased enzyme activity was previously reported for DNMT1 containing G589A or V590F mutations. [25]. Collectively, this evidence demonstrates that ADCA-DN-linked RFTS mutations disrupt normal autoinhibition of DNMT1, resulting in hyperactive enzymes. In contrast, studies examining HSAN1E-associated mutations have reported decreased activity in the mutant enzymes [17,41]. The HSAN1E-linked mutations investigated in these studies have been shown to induce misfolding of the RFTS domain [17]. It is possible that decreased protein stability could account for the reduced activity reported. Alternatively, HSAN1E-associated mutations could have different impacts on DNMT1 function than ADCA-DN-associated mutations. Thorough biochemical investigation of all disease-associated mutations is necessary to fully understand the impact of each mutation on DNMT1.

CD investigations revealed that mutant RFTS-containing DNMT1 had decreased thermal stability relative to its wild-type counterpart, while the relative stability of the RFTS domain was unaffected by the mutation. Moreover, the CD spectrum of the A554V RFTS domain was very similar to that of wild type, indicating that the mutation did not significantly affect the structure of the isolated domain. A554 is found in the helical bundle at the C-terminus of the RFTS domain (Figure 1b). The interaction between the C lobe of the RFTS domain and the methyltransferase domain is stabilized by a collection of interdomain hydrogen bonds. While A554 is not found directly at this interface, this residue is positioned nearby. The side chain of A554 points into the hydrophobic core of the C lobe of the RFTS domain [9,10]. Increasing the size of this side chain by replacement with valine could disrupt the alignment of nearby interdomain stabilizing hydrogen bonds, weakening normal autoinhibition without significantly impacting the structure of the domain, consistent with the data presented here. Interestingly, in recent work examining allostery in DNMT1 using activity-based CRISPR scanning, the C lobe of the RFTS domain was identified as a mutational hotspot. Biochemical analysis of several mutant enzymes showed that mutations in this region increased DNMT1 activity. The authors suggested that this increase in activity is due to loss of the inhibitory interdomain interaction typically present in DNMT1 [42].

**Figure 7.**
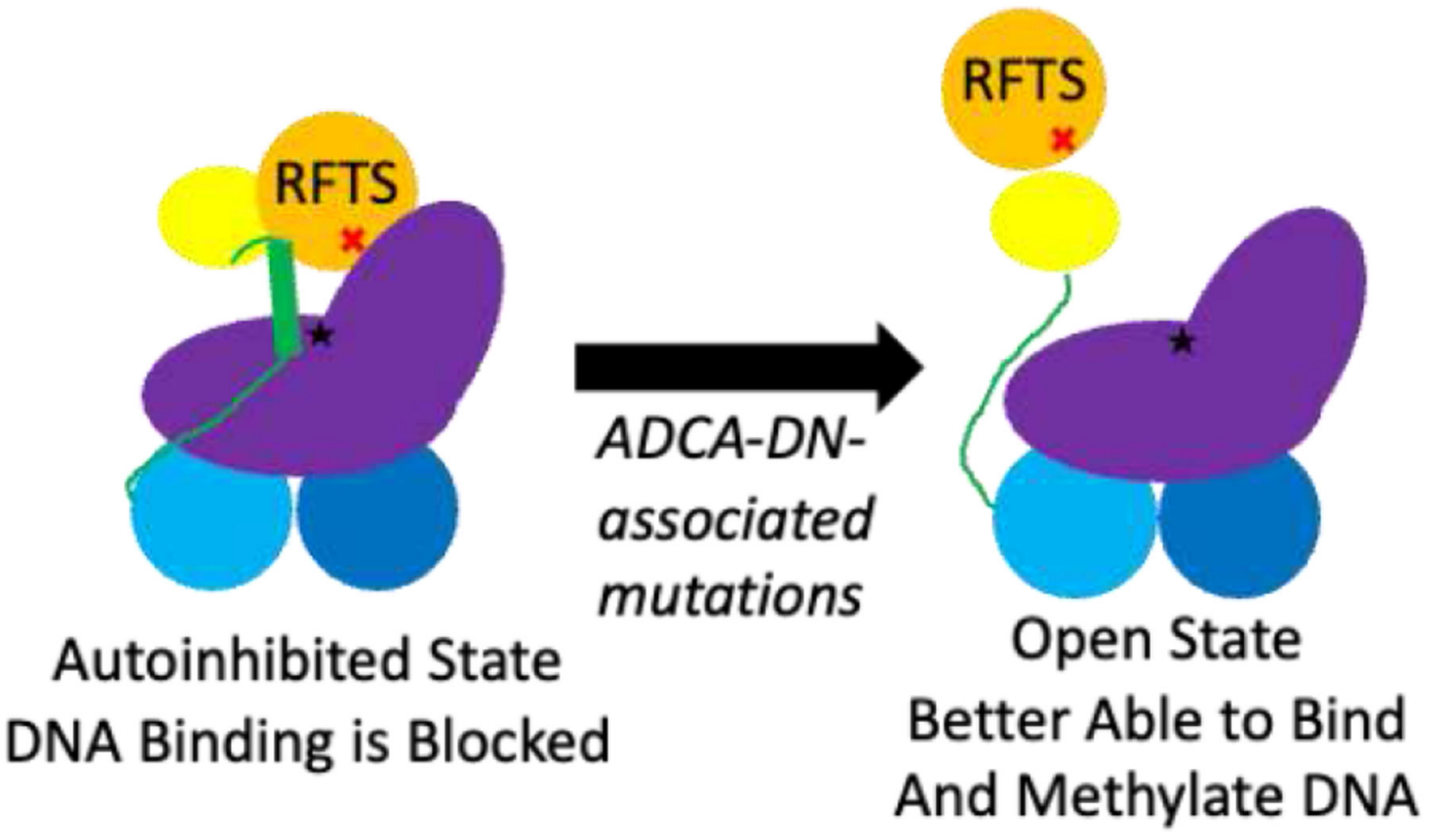
Model of disruption of RFTS-mediated autoinhibition. Usually, the C lobe of the RFTS domain makes interdomain interactions with the methyltransferase domain, blocking the DNA binding site (black star). ADCA-DN mutations like A554V (red X) weaken this interdomain interaction, allowing the enzyme to exist in an open, uninhibited state. This change in conformation induces a helix-to-loop transition of the autoinhibitory linker. Protein domains are colored according to Figure 1a.

While the CD spectra of wild-type and mutant RFTS domain were very similar, noticeable differences were observed in the RFTS-containing DNMT1 proteins. Secondary structure analysis suggests that introduction of A554V results in a ~2% loss in helical content in DNMT1. Since the same change in secondary structure content was not observed in the purified RFTS domains, we suggest that this change in structure comes from a region outside of the RFTS domain itself. The RFTS domain is near a region of DNMT1 termed the autoinhibitory linker (Figure 1a). Remarkably, this linker region adopts different conformations in different crystal structures of DNMT1. In the RFTS autoinhibited state, the linker adopts a helical conformation (Figure 1b). However, when the RFTS domain is absent, the linker adopts an extended loop conformation [3]. Presumably, as mutant DNMT1 transitions from an autoinhibited state to an open, active state, this linker region would undergo a conformational change (Figure 7). The helix-to-loop transition of the autoinhibitory linker could be responsible for the loss of helical content observed in the hyperactive mutant enzyme.

Methylation analysis of individuals with ADCA-DN showed increased DNA methylation. Normally unmethylated promoters and CpG islands were shown to be methylated in affected individuals. In addition, hypermethylated gene bodies and intergenic regions were further methylated in ADCA-DN patients [24]. Hyperactive DNMT1 in affected individuals could help explain these observed increases in DNA methylation. While there is ample evidence that ADCA-DN mutations at least partially relieve RFTS-mediated autoinhibition of DNMT1, it is possible for these mutations to also impact other RFTS functions. Further work examining the impact of these mutations on critical protein–protein regulatory interactions is crucial to fully understand the consequences of the mutations. The RFTS domain interacts with UHRF1 [12,13] and histone H3 tails [14-16]. The disease-associated RFTS mutations could disrupt or enhance these critical binding interactions, leading to mislocalization of hyperactive DNMT1 in cells. Detailed biochemical analysis of the impact of ADCA-DN-associated mutations on these binding interactions is an important future step. Our understanding of the molecular mechanisms that underlie ADCA-DN development would be greatly aided by a thorough understanding of how the RFTS mutations affect DNMT1 function.

## Supplementary Material

Supplement_DNA_Switzer

## Figures and Tables

**Table 1.** Secondary structure content of wild-type and A554V RFTS domain.

Domain	Helix ^1^	Antiparallel	Parallel	Turn	Other
Wild type	17.5	23.3	0	14.9	44.3
A554V	17.3	22.3	0	14.9	45.2

1 Analysis completed using BeStSel (http://bestsel.elte.hu) [27,28]. RMSD ≤ 0.043 for both fits.

**Table 2.** Secondary structure content of wild-type and A554V RFTS-containing DNMT1.

Protein	Helix ^1^	Antiparallel	Parallel	Turn	Other
Wild-type	16.1	26.4	0	14.1	43.4
A554V	14.4	26.5	0	14.4	44.7

1 Analysis completed using BeStSel (http://bestsel.elte.hu) [27,28]. RMSD ≤ 0.036 for both fits.

## Data Availability

The data presented in this study are available in this article and the Supplementary Materials.
