# Supplementary material for: Disease-Associated Mutation A554V Disrupts Normal Autoinhibition of DNMT1
Source: DNA (Basel). Author manuscript; Available in PMC 2023 Sep 1. (PMC10470860; doi:10.3390/dna3030010)
Supplement: Supplement_DNA_Switzer [file NIHMS1921666-supplement-Supplement_DNA_Switzer.pdf]

Article

Disease-associated Mutation A554V Disrupts Normal Autoinhibition of DNMT1

Rebecca L. Switzer [1,*], Zach J. Hartman [2], Geoffrey R. Hewett [3] and Clara F. Carroll [1]

[1] Department of Chemistry, Bucknell University, Lewisburg, PA 17837, USA
[2] Department of Biology, Bucknell University, Lewisburg, PA, 17837, USA
[3] Program in Cell Biology/Biochemistry, Bucknell University, Lewisburg, PA 17837, USA
* Correspondence: rebecca.switzer@bucknell.edu

Supplementary Materials

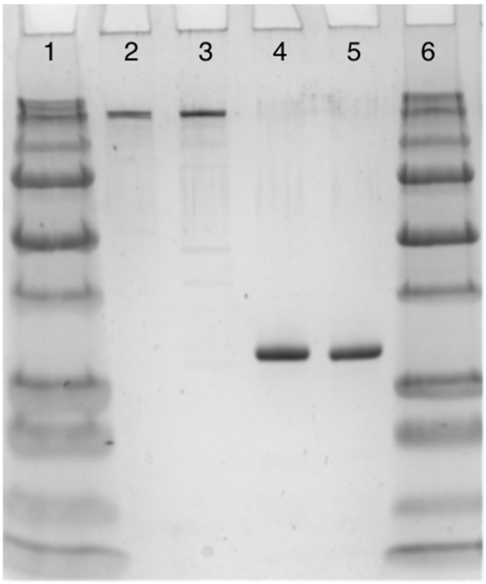

Figure S1. SDS-PAGE of purified proteins and domains. Roughly 0.5 μg of RFTS-containing DNMT1 proteins and 1.0 μg of RFTS domains were separated on a 12% TGX FastCast acrylamide gel (Bio-Rad) and stained using GelCode Blue (Thermo Fisher Scientific). Lanes 1 and 6: Bio-Rad Precision Plus Protein All Blue Prestained Ladder; Lane 2: wild-type RFTS-containing DNMT1; Lane 3: A554V RFTS-containing DNMT1; Lane 4: wild-type RFTS domain; Lane 5: A554V RFTS domain.

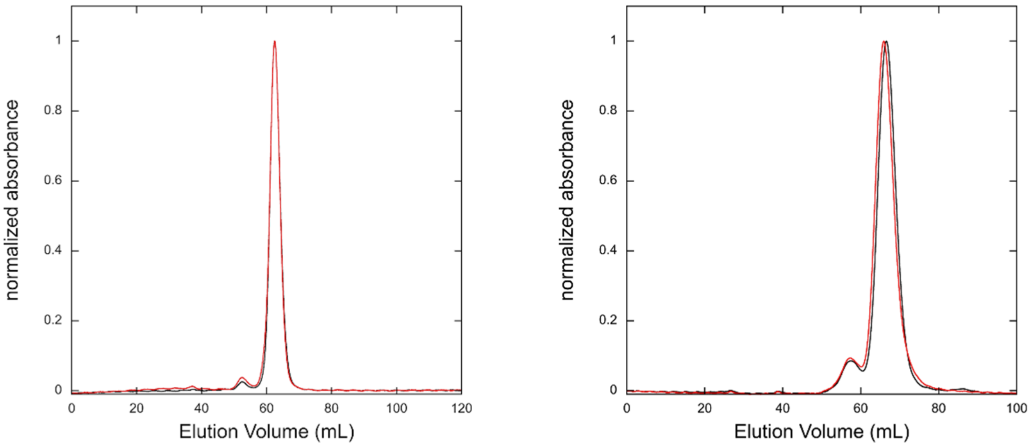

(**a**) (**b**)

Figure S2. Size-exclusion chromatograms of purified proteins and domains. (**a**) Purified wild-type (black) and A554V (red) RFTS domain was run on a Superdex 75 column. Both proteins elute as a single peak with a retention volume of 62.5 mL, indicating the mutation did not significantly impact the structure of the domain. (**b**) Purified wild-type (black) and A554V (red) RFTS-containing DNMT1 was run on a Superdex 200 column. Both proteins elute with a major single peak. The retention volume for the wild-type protein was 66.6 mL while the mutant protein eluted slightly earlier with a retention volume of 65.9 mL, suggesting the mutation resulted in a slight structural change in the larger protein. Absorbance values were normalized for visualization.

Table S1. Replicate melts of the RFTS domain by CD.

**Protein**	**Wavelength**	**Observed T_m (°C)[1]**
Wild-type	198 nm	47.0 ± 0.1
Wild-type	198 nm	47.0 ± 0.1
Wild-type	198 nm	46.8 ± 0.1
Wild-type	200 nm	46.9 ± 0.2
A554V	198 nm	47.0 ± 0.1
A554V	198 nm	46.7 ± 0.1
A554V	198 nm	47.0 ± 0.1
A554V	200 nm	47.0 ± 0.1

[1]Melting curves were fit to the Boltzmann equation to determine the observed T_m.

.

Table S2. Replicate melts of RFTS-containing DNMT1 by CD.

Protein	Wavelength	Observed T_m (°C)[1]
Wild-type	207 nm	55.0 ± 0.1
Wild-type	207 nm	54.9 ± 0.1
Wild-type	207 nm	55.1 ± 0.1
Wild-type	198 nm	55.3 ± 0.3
A554V	207 nm	53.2 ± 0.1
A554V	207 nm	53.4 ± 0.1
A554V	207 nm	53.2 ± 0.1
A554V	198 nm	53.3 ± 0.5

[1]Melting curves were fit to the Boltzmann equation to determine the observed T_m.

.

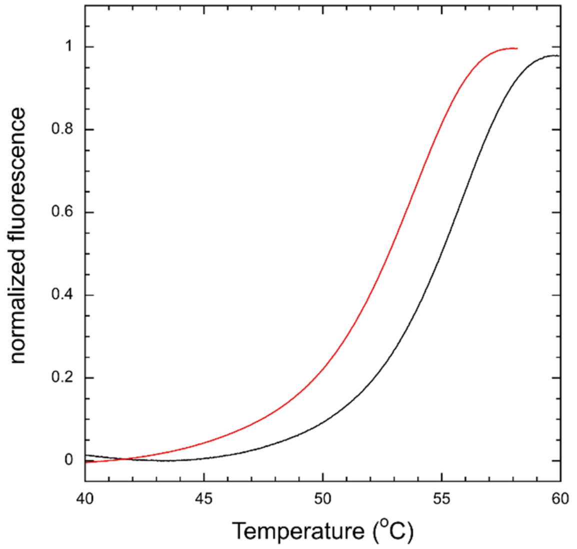

Figure S3. Differential scanning fluorimetry of RFTS-containing DNMT1. Representative melting curves for wild-type (black) and A554V (red) RFTS-containing DNMT1. SYPRO Orange fluorescence has been normalized for visualization. Fitting the melting curves to the Boltzmann equation yields observed T_m values of 55.2 ± 0.1 °C and 53.2 ± 0.1 °C for wild-type and A554V RFTS-containing DNMT1, respectively.
